# Artificially Increased Yolk Hormone Levels and Neophobia in Domestic Chicks

**DOI:** 10.3390/ani5040408

**Published:** 2015-11-30

**Authors:** Aline Bertin, Cécile Arnould, Chantal Moussu, Maryse Meurisse, Paul Constantin, Christine Leterrier, Ludovic Calandreau

**Affiliations:** 1 Physiologie de la Reproduction et des Comportements, Institut National de la Recherche Agronomique (INRA), UMR85, Nouzilly 37380, France; E-Mails: cecile.arnould@tours.inra.fr (C.A.); chantal.moussu@tours.inra.fr (C.M.); maryse.meurisse@tours.inra.fr (M.M.); paul.constantin@tours.inra.fr (P.C.); christine.leterrier@tours.inra.fr (C.L.); ludovic.calandreau@tours.inra.fr (L.C.); 2 Centre National de la Recherche Scientifique (CNRS), UMR7247, Nouzilly 37380, France; 3 Université François Rabelais de Tours, Tours 37000, France; 4 Institut Français du Cheval et de l’Equitation (IFCE), Nouzilly 37380, France

**Keywords:** yolk hormones, maternal effects, neophobia, fear, behavioral development

## Abstract

In birds there is compelling evidence that the development and expression of behavior is affected by maternal factors, particularly via variation in yolk hormone concentrations of maternal origin. In the present study we tested whether variation in yolk hormone levels lead to variation in the expression of neophobia in young domestic chicks. Understanding how the prenatal environment could predispose chicks to express fear-related behaviors is essential in order to propose preventive actions and improve animal welfare. We simulated the consequences of a maternal stress by experimentally enhancing yolk progesterone, testosterone and estradiol concentrations in hen eggs prior to incubation. The chicks from these hormone-treated eggs (H) and from sham embryos (C) that received the vehicle-only were exposed to novel food, novel object and novel environment tests. H chicks approached a novel object significantly faster and were significantly more active in a novel environment than controls, suggesting less fearfulness. Conversely, no effect of the treatment was found in food neophobia tests. Our study highlights a developmental influence of yolk hormones on a specific aspect of neophobia. The results suggest that increased yolk hormone levels modulate specifically the probability of exploring novel environments or novel objects in the environment.

## 1. Introduction

The reluctance to approach novel objects or novel food resources is observed in a large range of *taxa* and is commonly called neophobia. Neophobia is particularly well described in wild bird populations. It plays a key role in habitat selection, feeding innovation and the capacity to adapt to novel environments or novel resources [1,2]. Neophobia can be defined as the avoidance of an object or other aspect of the environment solely because it has never been experienced and is dissimilar from what has been experienced before. This reaction is associated with fear and the physiological and behavioral correlates of fear responses [3]. 

Despite domestication, neophobia is also observed in domestic hens (*Gallus gallus domesticus*). In laboratory conditions, this emotional reaction is described as a brief response followed by investigation of the novel food or object ([4] for a review). However, in commercial conditions, incapacity to overcome neophobia and accept novel foods is reported [5]. Domestic hens usually receive food in the form of a complete diet. During the course of development, feed ingredients in the complete diet are changed to meet specific nutritional needs. These changes alter the physical characteristics of feed (e.g., color, odor) in a way that is barely perceptible to humans; however, domestic birds have an acute sensory perception of feed. Birds exhibit neophobic responses when a single sensorial property of their food is changed [6,7]. Moreover, the amplitude of neophobic responses is enhanced when multiple sensorial properties of food are changed simultaneously. For example, the visual and tactile properties of food interact and potentiate the reaction of animals towards novel odors [8]. Thus, under commercial conditions, they sometimes express strong and damaging neophobic responses when facing such changes [9]. The refusal to accept new foods leads to a major reduction in feed intake and, subsequently, in growth and animal welfare [10]. In addition to the expression of fear, food neophobia reduces sanitary conditions with pecking redirected toward litter or feces [11]. For example, turkeys decrease feed intake and increase exploratory behavior upon receiving an unknown feed [12]. The limited research on food neophobia in poultry has focused mainly on how to “deactivate” this behavior at an early age [13]. Currently little is known about early developmental programming effects on this behavior. Evidence for a quantitative genetic basis for different levels of neophobia grew with the variation found between domestic and wild type birds [6,14] and the possibility to divergently select for the capacity to incorporate a novel food (novel insect prey) into the diet (Japanese quail, *Coturnix coturnix japonica*, [15]). 

On the other hand, many studies draw attention to the role of developmental environments in shaping the behavior of young domestic birds. In particular, yolk hormones of maternal origin may adjust offspring phenotype according to environmental conditions. In Japanese quail, the environment provided to mothers (human-animal relationship, presence of a shelter, social instability, physical stress) during egg formation engender significant variations in yolk steroid concentrations (testosterone, androstenedione, progesterone). Although the direction of the effects is not predictable, the general emotional reactivity of offspring, including fear responses toward novel objects or humans, was consistently found to be influenced by these hormonal effects [16,17,18,19]. We also found evidence of a link between yolk hormone levels and inherent fearfulness (*i.e.*, propensity to express fear responses) in selected lines of quail. Higher yolk hormone concentration levels were found in eggs of the low inherent fearfulness line compared to eggs of the high inherent fearfulness line. An impact (positive or negative) of maternal androgens on the behavior and body mass of offspring has also been shown by administering physiological doses of hormones (androstenedione, testosterone) into eggs [20,21,22,23].

Far less is known about the implication of yolk hormone levels in the development of fear related behaviors in domestic hens. Janczak *et al.* [24] reported that maternal stress (feed restriction) increased offspring’s duration of tonic immobility which indicated increased fearfulness. On the contrary, artificial maternal corticosterone elevation during egg formation has been reported to decrease offspring’s duration of tonic immobility and slightly increase activity in an open-field test [25], which indicates lower fearfulness. So far, most studies conducted on domestic chickens focus on the implication of maternal corticosterone levels on offspring’s physiology and behavior [26] for a review]. Maternal stress such as unpredictable food access [27] or early experience of social isolation [28] were found to alter the expression of stress-specific genes or genes related to immunity in the offspring. However, as mentioned by the authors, the implication of yolk gonadal hormones remains to be elucidated. It is difficult to identify the pathway of maternal effects. Indeed, maternal stress or increased maternal corticosterone levels also alter other factors such as egg mass [25], which could a play part in the construction of offspring phenotype. 

In a previous study we observed a decrease in egg mass and a significant increase in the yolk concentrations of estradiol, testosterone and progesterone in Leghorn hens exposed to moderate heat stress whereas maternal corticosterone levels were not affected [29]. Our hypothesis was that such variations in yolk hormone concentrations could influence body mass and play a part in the propensity to express neophobia in young chicks independently of egg mass. Therefore we experimentally enhanced yolk hormone concentrations prior to incubation and characterized the immediate reaction of chicks to novel items (food, object and environment). 

## 2. Experimental Section

### 2.1. Ethics Statement

All birds were maintained at the Experimental Unit PEAT of INRA (Nouzilly, France). The Experimental Unit is registered by the ministry of Agriculture with the license number B-37-175-1 for animal experimentation. All experiments were approved by the Animal Ethics Committee Val de Loire, CEEA Vdl (permit number 2012-10-6). All experiments were performed in accordance with the European Communities Council Directive 2010/63/UE. All animals were rehabilitated at the end of the experiment.

### 2.2. Egg Injections 

One hundred and forty-five eggs from White Leghorn layers were provided by the experimental unit PEAT of INRA (Nouzilly, France). Two groups were considered: a group of 70 sham control eggs injected with a vehicle solution (C group) and a group of 75 eggs injected with the hormonal solution (H group). Eggs were selected so that the mass of the eggs did not differ significantly between the groups (Mean ± SE, C group: 56.88 ± 0.4 g; H group: 56.9 ± 0.3 g, F_1,__143_ = 0.001, *p* = 0.97). 

In a previous study we demonstrated that a moderate maternal environmental challenge (moderate heat stress) significantly increased the yolk progesterone (P4), testosterone (T) and estradiol (E2) concentrations in Leghorn eggs but decreased the egg mass [29]. To simulate this increase in yolk hormone levels, we injected H eggs prior to incubation with a hormonal solution containing a mixture of P4, T and E2. We chose to increase the overall yolk concentrations by calculating the difference observed between the control population and the challenged population described by Bertin *et al.* [29]. For example, P4 concentrations were found to be 1823.7 ng/g in the control egg yolks *versus* 2181.9 ng/g in the yolks of the challenged population (which means a difference of 358.2 ng/g). The mean yolk mass was determined with 30 additional eggs (17.8 ± 0.3 g). We used the difference between the two observed concentrations and yolk mass (358.2 × 17.8) to determine the quantity to be injected into each egg. Therefore each egg was injected with 6376 ng of P4, 6.76 ng of T and 16.02 ng of E2 dissolved in 50 μL of vehicle (corn oil, Sigma Life Science). 

Artificial increase of yolk hormones was chosen in order to focus specifically on hormone-mediated maternal effects rather than maternal effects induced by changes in egg mass or the general quality of eggs subsequent to maternal challenge. Artificially increasing yolk hormones prior to incubation is commonly used in birds to examine the short- and long-term effects of yolk hormones on the development of phenotypes [30]. Before injection, all eggs were carefully cleaned and disinfected with 70% ethanol and a hole was bored into the eggshell above the air sac using a 25-G drill. The solution was delivered to the yolk using a 100-μL Hamilton syringe mounting a 25-G sterile needle. As fully described by Bertin *et al.* [23], the injection hole was then sealed by gluing a tiny piece of cleaned and disinfected eggshell to the egg immediately after injection. The eggs were left undisturbed for one hour and then placed in alternative rows on each shelf of two incubators maintained at 37.8 °C and 56% relative humidity with automatic and continuous turning. On day 14 of incubation, non-fertile eggs or eggs containing prematurely dead embryos were eliminated (12 for C eggs and 25 for H eggs). Three days before hatching, the rotation was stopped and the temperature was reduced to 37.6 °C. 

Artificial increase of yolk hormones was chosen in order to focus specifically on hormone-mediated maternal effects rather than maternal effects induced by changes in egg mass or the general quality of eggs subsequent to maternal challenge. Artificially increasing yolk hormones prior to incubation is commonly used in birds to examine the short- and long-term effects of yolk hormones on the development of phenotypes [30]. Before injection, all eggs were carefully cleaned and disinfected with 70% ethanol and a hole was bored into the eggshell above the air sac using a 25-G drill. The solution was delivered to the yolk using a 100-μL Hamilton syringe mounting a 25-G sterile needle. As fully described by Bertin *et al.* [23], the injection hole was then sealed by gluing a tiny piece of cleaned and disinfected eggshell to the egg immediately after injection. The eggs were left undisturbed for one hour and then placed in alternative rows on each shelf of two incubators maintained at 37.8 °C and 56% relative humidity with automatic and continuous turning. On day 14 of incubation, non-fertile eggs or eggs containing prematurely dead embryos were eliminated (12 for C eggs and 25 for H eggs). Three days before hatching, the rotation was stopped and the temperature was reduced to 37.6 °C. 

### 2.3. Housing Conditions 

We kept 70 chicks (40 C and 30 H chicks) that all hatched on the 21st day of incubation. Each chick was identified with a numbered ring on its leg. The 70 chicks were placed in pairs from the same treatment in wire-covered plastic tubs (50 cm × 40 cm × 30 cm; length × width × height) with wood shavings on the floor. The pairs of chicks were equally allocated to two rooms. Chicks were maintained in an 11 h light/13 h dark cycle, with water and food available *ad libitum*. The chicks were fed with a conventional starter mash (PEAT, INRA Val de Loire, France) dispensed in feeding troughs (length 50 cm). The troughs were covered with a metallic roof containing 12 circular holes (diameter, 5 cm); these holes provided the chicks with sufficient access to the feed while avoiding food spillage. Two opaque drinking bottles (1 L) were placed in each cage. The ambient temperature was maintained at 33 ± 1 °C from hatching until the chicks were 8 days old, after which it was decreased by 1 °C per day. The sex of each chick was determined by comb size at 3 weeks of age. The control group was composed of 20 females and 20 males, and the H group was composed of 13 females and 17 males. 

### 2.4. Growth of Chicks

The chicks were weighed at hatching (day 1) and when they were 3 days, 10 days, 17 days and 25 days old. 

### 2.5. Characterization of Neophobia

#### 2.5.1. Food Neophobia

All of the tests used have previously been described by Bertin *et al.* [31]. The chicks are extremely distressed when isolated, and therefore we simultaneously tested two chicks from the same exposure conditions (and the same pen) in all of the tests. Chicks have a very rapid growth rate, therefore, all birds were tested for each test at the same age. This design also controlled for the time during which chicks were in contact with their familiar standard food before each test. We used short-term 3-minute tests to assess the immediate reaction to novel foods. We tested the effect of treatment when chicks were exposed to a change in the olfactory properties of their familiar food (single sensorial modality, Test 1) and a change in multiple sensorial properties of the food (raw cereals). We used cracked corn-wheat (cereals contained in familiar pellets, Test 2) and millet seeds (totally unknown to the birds, Test 3).

For the three tests, each pair of chicks (from the same treatment) was placed in a testing cage, similar to their home cage, after 1 h 30 min of food deprivation. The chicks were transported in a 15 cm × 15 cm × 15 cm container and then deposited and held blind in an enclosure (20 cm × 6 cm × 20 cm) placed at the opposite side of the feeding trough (identical to the familiar trough). After 30 s, the chicks were released and an observer hidden behind a curtain with small observation windows recorded the behavior of one focal bird of each pair (20 pairs of C chicks and 15 pairs of H chicks) for a 3-minute period. Focal birds were chosen randomly beforehand and identified by a blue-colored mark placed on their heads. The experimenter recorded the latency to eat the food and the time spent eating each food (the bird was considered to be eating when mandibulation and neck and throat movements caused by swallowing were observed. Eating behavior was considered to stop when the bird removed its head from the hole of the feeder and no more swallowing was observed for at least 3 s). In Test 1 (4 days of age), the trough contained 200 g of the familiar starter mash odorized with a mixture of artificial scents known to be perceived by chicks. We used a mix of isoamyl acetate (0.03 g), vanilline (0.03 g), and strawberry (0.03 g) in the form of a translucent powder. In Test 2 (7 days of age) we used 200 g of cracked corn-wheat, and in Test 3 (8 days of age) we used 200 g of millet seeds.

#### 2.5.2. Object Neophobia

The protocol was similar to the novel food tests except that the familiar trough was replaced by an unfamiliar green and yellow plastic trough filled with 200 g of the familiar food. The experimenter recorded the latency to eat the food and time spent eating for a 5-minute period. This test was carried out at 18 days of age. 

#### 2.5.3. Fear of a Novel Environment

To assess fear of a novel environment we used a classical open-field test. This test involves transferring the birds from the familiar home cage to a new, open, larger and more lighted environment. The chicks were individually placed in the middle of an open cylindrical arena (diameter, 120 cm; height, 35 cm) on a linoleum floor for 5 min. To assess their locomotor activity, two perpendicular 120-cm-long lines were drawn in the arena, dividing it into four equal parts. The latency of the first step and the activity of the birds (number of times a bird crossed a line) were recorded by the experimenter hidden behind a curtain with observation windows. The chicks were tested when they were 15–16 days old.

### 2.6. Statistical Analyses

We compared the number of chicks hatched out of the fertile eggs using chi-square tests. The mass of chicks was analyzed using ANOVA for repeated measures (age) with treatment and sex as main factors and *post hoc* protected least significant difference Fisher tests. 

Behavioral data did not follow a normal pattern of distribution; therefore, the non-parametric Mann-Whitney *U*-test was used for intergroup comparisons. Friedman and Wilcoxon tests were used for intra-group comparisons. We evaluated the intra-individual consistency to approach novel foods and the correlation between novel foods and novel object tests. Therefore we used Spearman rank correlations to correlate the latencies to eat in the different tests. Sex effects are mentioned only when significant. Data are presented as the mean ± standard error (SE). All analyses were performed using the StatView software (SAS Institute Inc., Cary, NC, USA), with significance accepted at *p* ≤ 0.05.

## 3. Results

### 3.1. Hatching Success and Mass of Chicks 

No significant effect of egg treatment was observed on hatching success (C group: 42 chicks hatched out of 58 fertile eggs; H group: 31 chicks hatched out of 50 fertile eggs, Chi2 = 0.89, *p* = 0.34). 

We found no significant effect of the treatment on the mass of chicks (Table 1, ANOVA on repeated measures, F_1,65_ = 0.27, *p* = 0.60). There was a significant effect of sex but no interaction between sex and treatment (ANOVA, sex effect: F_1,65_ = 14.25, *p* < 0.01; sex × treatment: F_4,260_ = 0.14, *p* = 0.96). The mass of males was higher than the mass of females. 

**Table 1:** Mean mass (± SE) of C (*n =* 40) and H chicks (*n =* 30) at 1, 3, 10, 17 and 25 days of life.

	Mass (g)				
Groups	day 1	day 3	day 10	day 17	day 25
C chicks	40.2 ± 0.45	41.1 ± 0.71	84.8 ± 1.73	162.5 ± 3.26	269.1 ± 5.41
H chicks	39.2 ± 0.49	40.5 ± 0.6	84.7 ± 1.45	162.7 ± 2.93	267.6 ± 5.49

### 3.2. Characterization of Neophobia

#### 3.2.1. Food Neophobia 

We found no significant difference between C chicks and H chicks in the latency to eat or time spent eating during tests with familiar odorized food or during tests with cracked corn-wheat or tests with millet seeds (Table 2). 

**Table 2:** Mean (± SE) latency to eat (s) and time spent eating (s) during food neophobia tests of C chicks (*n =* 20 pairs) and H chicks (*n =* 15 pairs).

Food Neophobia Test	Parameters Measured	C Chicks	H Chicks	* *p*-Value
Odorized familiar food Test 1	latency to eat (s)	72.1 ± 10.9	54.1 ± 10.1	0.48
	time spent eating (s)	41.7 ± 6	46.4 ± 6.3	0.87
Corn wheat mix Test 2	latency to eat (s)	75.3 ± 15.2	63.8 ± 15.5	0.48
	time spent eating (s)	11.6 ± 2.5	9.6 ± 2.1	0.23
Millet seeds Test 3	latency to eat (s)	132.7 ± 15.5	135.1 ± 13.1	0.73
	time spent eating (s)	5.4 ± 1.9	13.0 ± 5.0	0.75

* Mann-Whitney U-tests *p* value.

Within both groups, the latencies to eat differed significantly according to the type of food (Friedman, *p* < 0.01). Within both groups, chicks took longer to eat the millet seeds than to eat the cracked corn-wheat or the familiar odorized food (Table 2, Wilcoxon, *p* < 0.01 for all comparisons). During the test with millet seeds, 18 pairs of chicks refused to eat (nine controls and seven H). The latency to eat the familiar odorized food or the corn-wheat did not differ significantly within both groups (Wilcoxon, *p* > 0.05 for all comparison).

Independent of the treatment, the latency to eat the familiar odorized food was significantly correlated with the latency to eat the cracked corn-wheat (*r =* 0.58, *p* < 0.01) but not with the latency to eat the millet seeds (*r =* 0.27, *p* = 0.11). 

#### 3.2.2. Object Neophobia 

**Figure 1:**
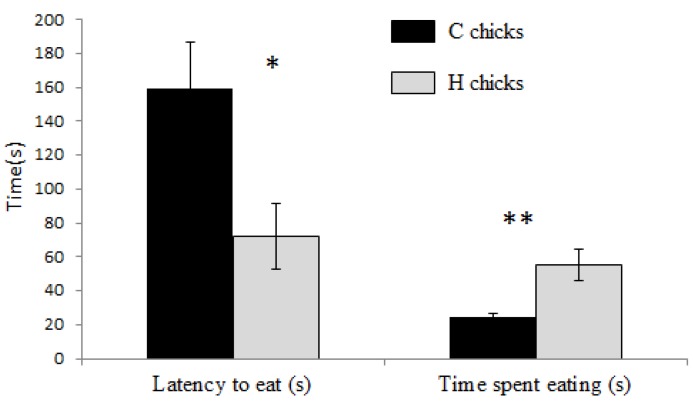
Mean ± SE latency to eat (s) and time spent eating (s) in the novel object test in C chicks (*n =* 20) and H chicks (*n =* 15). * Mann-Whitney U-test, *p* < 0.05; ** *p* < 0.01.

The latency to eat was significantly shorter in H chicks than in C chicks in the novel-object test (Figure 1). The time spent eating was significantly higher in H chicks than in C chicks. 

The latency to eat during the novel object test did not correlate significantly with the latency to eat the familiar odorized food (*r =* 0.03, *p* = 0.83) nor with the latency to eat the cracked corn-wheat (*r =* 0.1, *p* = 0.49) or the millet seeds (*r =* 0.1, *p* = 0.41).

#### 3.2.3. Fear of a Novel Environment

The number of lines crossed and the number of distress calls were significantly higher in H chicks than in C chicks (Table 3). The latency of first step did not differ significantly.

**Table 3:** Mean (± SE) latency of first step, number of lines crossed and number of distress calls in C chicks (*n =* 40) and H chicks (*n =* 30) in the open-field test.

Parameters Measured	C Chicks	H Chicks	* *p*-Value
latency of first step (s)	76.3 ± 13.0	47.8 ± 7.93	0.18
number of distress calls	183.5 ± 14.7	227.1 ± 13.7	0.02
number of lines crossed	9.8 ± 1.47	15.0 ± 1.94	0.01

* Mann-Whitney U-tests *p-*value.

## 4. Discussion 

The aim of this study was to assess whether the propensity of chicks to express neophobia could be shaped by yolk hormone levels. When the maternal population is exposed to a stress, a combination of yolk hormones is commonly affected [16,17,27,28,29]. As a consequence, we did not focus on the action of a specific yolk hormone, as our aim was to mimic the consequences of a maternal stress. Our results showed that chicks exposed *in ovo* to elevated concentrations of estradiol, testosterone and progesterone (simulating a maternal challenge) expressed significantly less fear of a novel object or a novel environment but did not differ from controls regarding food neophobia. 

We did not find significant effects of the treatment on latencies to eat or time spent eating each type of food across three testing conditions with different degrees of novelty. This result suggests that yolk hormone levels did not shape the expression of food neophobia specifically. In accordance with our finding in zebra finches (*Taeniopygia guttata*) from eggs with artificially increased testosterone levels that did not differ from controls in their latencies to approach and eat a novel food during their first encounter [32]. Similarly, in canaries (*Serinus canaria*), increased levels of yolk testosterone did not form the main source of variation in the expression of food neophobia [33]. In a previous study, we observed a significant increase in yolk concentrations of estradiol, testosterone and progesterone in Leghorn hens exposed to moderate heat challenge. In comparison with control chicks, offspring from these hens expressed significantly fewer distress calls when facing a novel food, but the latencies to approach and time spent eating were not affected [29]. No distress calls were emitted in the present study, and as maternal challenges modify other properties of the egg (*i.e.*, yolk mass), it remains difficult to attribute the effect on chick vocalization solely to variation in yolk hormone levels. 

The significant correlation between the latency to eat the familiar odorized food and the mix of corn wheat (cereals contained in the familiar pellet food) suggests a consistency in responses when foods presented a moderate degree of novelty. Additional work is needed to determine whether food neophobia could be considered a temperament trait in domestic hens, which is suggested in populations of House sparrows (*Passer domesticus*) [34]. We did not find a correlation between the latency to eat the millet seeds and the latencies to eat other types of food, but this lack of correlation could be due to a ceiling effect caused by the short duration of our test and the maximum score (180s) attributed to pairs of chicks refusing to eat the millet seeds. 

In contrast to food neophobia tests, very clear effects of the treatment were observed in the novel object test and in the open-field test. This result draws attention to the necessity of considering food neophobia on one hand, and object neophobia and fear of novel environment on the other hand as distinct processes. H chicks approached the novel object significantly faster and spent more time eating than controls, suggesting a reduced emotional response in H chicks compared to controls. Similarly, quail chicks from eggs with increased T levels approached a novel object faster than controls [20], suggesting a more “proactive” profile. However, discrepancies of yolk hormone effects are found in latencies to approach novel objects, as reviewed by Vergauwen *et al.* [33]. Depending on the species and the dose injected, shorter, longer or no effect on latencies to approach a novel object are all observed following increased yolk hormone levels (T or T+A4). We also found no correlation between the latency to approach a novel object and the latency to approach novel foods. In the very few studies investigating the influence of yolk hormones on both food and object neophobia this absence of correlation was also observed [32,33], suggesting two independent behavioral traits in adult birds. In the present study the tests were conducted at different ages and on young birds. As fearfulness changes during development [35] we cannot rule out the possible effect of age. 

When tested individually in the open-field test, higher levels of activity (number of lines crossed and number of distress calls) were observed in H chicks compared to controls. In poultry species, fear is associated with behavioral inhibition characterized by both reduced vocalization and motor activity [36]. Quiet and inactive animals are commonly considered to have a higher level of emotional reactivity than active animals [37]. H chicks thus appeared less fearful in the novel environment. Increased levels of activity in novel environments with increased yolk T levels were also found in the pied flycatcher (*Ficedula hypoleuca*) [38]. In the Japanese quail, variation in yolk hormonal contents (both correlational and experimental) was almost consistently found to influence locomotor activity in open-field tests, but with no consensus concerning the direction of the effect [17,18,19,20,21,39,40]. Hegyi and Schwabl [22] have argued that different yolk androgens interact and differentially affect offspring phenotypes. For example, the authors reported that an injection of A4 in the yolk increased the locomotor activity in the open-field test but had no effect on the mass of quail chicks, whereas T injection reduced mass gain and had no effect on the open-field responses. In addition, non-linear dose response effects could also explain the discrepancy observed, as observed for nestling growth in starlings (*Sturnus unicolor*) [41]. Our results show a potential better ability to cope with novelty with increased yolk hormone levels. In the same direction, mothers with increased plasma corticosterone levels were found to produce offspring that were less fearful [25]. However, the mechanisms of action and potential detrimental trade-offs (e.g., immunity) remain to be elucidated.

In our study, no treatment effect was observed on chick mass or hatching success. Although more H eggs were discarded during incubation, it was difficult to determine whether the eggs were non-fertile or contained very early dead embryos. Yolk hormone levels show enhanced, reduced or no effect on growth of young birds in a large range of species ([41,42] for reviews). This lack of consistency highlights our current incomplete understanding of yolk hormones influences in interplay with other egg components and genetic factors. The present study suggests that there are no deleterious effects associated with elevated yolk hormone levels on the growth of chicks. 

## 5. Conclusions

Our results show that variations in yolk hormone levels modulate the propensity to explore a novel environment and novel objects in the environment. To our knowledge, this developmental influence has not yet been studied in domestic hens. Recent studies in domestic hens have all pointed out that adaptation of mothers to challenging environments translates into maternal effects and shapes the morphology and behavioral development of the offspring [28,29,30,31,32,33,34,35,36,37]. Currently, studies are scarce and leave a gap in knowledge about the nature of these maternal effects. The vast amount of maternal influences during prenatal development can affect the development of fear-related behaviors, which could impair the capacity of young chicks to cope with their environment. We therefore emphasize the importance of studying environmental influences on the early ontogenetic niche of chicks, which is constituted by the egg. 
